# Presence of bacteria in psoriatic lesions: A retrospective cohort study

**DOI:** 10.1016/j.jdin.2023.09.003

**Published:** 2023-10-02

**Authors:** Gitanjali Bhushan, Jocelyn Simmers, Alexandra Flamm

**Affiliations:** a Penn State College of Medicine, Hershey, Pennsylvania; b Department of Dermatology, Milton S. Hershey Medical Center, Hershey, Pennsylvania

**Keywords:** bacteria, dermatitis, dermatopathology, erosion, neutrophils, psoriasis, skin diseases

*To the Editor:* Atopic dermatitis (AD) and psoriasis are inflammatory skin diseases that negatively impact patients' quality of life.^1^^,^^2^ While it is known that AD flares can be triggered by bacterial colonization or infection of a lesion, particularly by Staphylococcus aureus, less is known about the bacterial yield seen in psoriasis and its flares.^3^ Although local colonization and infection of psoriasis is generally prevented by high antimicrobial peptides (AMPs), this can occur.^4^ Given this, we sought to further quantify the rates of bacterial colonies seen in psoriasis.

This study was approved by the Penn State institutional review board. A retrospective chart review was used to identify individuals >18 years with a biopsy of either psoriasis or eczematous dermatitis (ED). ED was defined as biopsies with a final diagnosis containing the phrase “spongiotic dermatitis” and a comment that included AD within the differential. The final diagnosis was confirmed by a dermatopathologist (A.F.) and demographic data and histologic parameters were collected including presence of bacteria, erosions, and presence of neutrophils in the stratum corneum. All survey data were presented as the number of subjects with percentages. Categorical and continuous variables were examined using the chi-square and Fisher exact tests as appropriate. Relative risk and odds ratios were reported with 95% confidence intervals. Data analyses were conducted in April to May 2022 using SAS 9.4 software (SAS Institute Inc).

Fifty patients with psoriasis and 51 patients with ED were evaluated (Table I). Psoriasis samples demonstrated erosion in 6% (*n* = 3), neutrophils in 98.8% (*n* = 49), and bacterial involvement in 18% of cases (*n* = 9). ED samples had higher rates of erosion (*n* = 15, 29.4%) and a similar rate of bacterial involvement (*n* = 9, 17.7%) when compared to psoriasis samples. There was a similar prevalence of bacteria in psoriasis (18.0%) and dermatitis (17.65%) samples (*P* < .5841). There was no difference in prevalence of bacteria by anatomic site (*P* < .9432) or age group (*P* < .9538, Table I).

**Table I:** Patient characteristics of psoriasis and dermatitis cohorts

	PS (*n* = 50)	Dermatitis (*n* = 51)	*P* value
Mean age ± SD, yr	51.24 ± 19.45	59.39 ± 18.96	
Female (%)	30 (60.0)	23 (45.0)	
Bacteria present (%)	9 (18.0)	9 (17.65)	<.5841
Erosion present (%)	3 (6.0)	15 (29.41)	<.9997
Neutrophils present (%)	49 (98.0)	21 (41.18)	<.0001
Anatomic site (%)			
Head and neck	5 (10.0)	2 (3.92)	
Upper extremities	8 (16.0)	20 (39.2)	
Lower extremities	17 (34.0)	9 (1.76)	
Torso	19 (38.0)	17 (33.3)	
Other/not specified	1 (2.0)	3 (5.88)	

+1 Bacteria population∗	PS (*n* = 9)	Dermatitis (*n* = 9)	*P* value
Anatomic sites of +1 bacteria			<.9432
Head and neck	0	0	
Upper extremities	0	5 (55.5)	
Lower extremities	4 (44.4)	1 (11.1)	
Torso	5 (55.5)	3 (33.3)	
Other/not specified	0	0	
Age of those with +1 bacteria			<.9538
0-17	1 (11.1)	0	
18-39	2 (22.2)	2 (2.22)	
40-59	4 (44.4)	3 (33.3)	
60-100	2 (22.2)	4 (44.4)	

*PS*, Psoriasis.

∗ Denominator is the 9 patients with +1 bacteria population in the PS cohort and the 9 patients with +1 bacteria in the dermatitis cohort. Bacterial load was quantified on a 1+ to 3+ scale, with 1+ denoting less 25% involvement of the stratum corneum/epidermal surface, 2+ signifying 25% to 75% involvement, and 3+ signifying greater than 75% involvement.

Pathophysiologically, psoriasis is thought to have lower rates of bacterial involvement due to the high levels of AMPs they harbor in comparison to dermatitis, however we found similar rates of bacterial involvement in psoriasis when compared to ED. In contrast, other studies comparing the two processes have found bacterial colonies at higher rate in AD when compared to psoriasis.^5^

Limitations of this study include small cohort size, that patients may have been actively treating their disease at time of biopsy, that biopsies were taken of ED which may not all represent true AD and these areas may not have had the typical clinical appearance of psoriasis and dermatitis. Additionally, due to the type of samples reviewed (skin biopsies without Gram stains), we were unable to fully characterize the bacterial species noted.

In conclusion, we found that histologically rates of bacterial involvement are similar between EDs and psoriasis biopsies, adding to the limited literature that psoriatic lesions indeed do experience bacterial involvement despite the anti-inflammatory role of AMPs. Further studies characterizing bacterial species in psoriatic lesions may help focus treatment recommendations.

## Conflicts of interest

None disclosed.
